# Non-Coding RNAs in Kidney Stones

**DOI:** 10.3390/biom14020213

**Published:** 2024-02-11

**Authors:** Guilin Wang, Jun Mi, Jiangtao Bai, Qiqi He, Xiaoran Li, Zhiping Wang

**Affiliations:** Department of Urology, Institute of Urology, Gansu Nephro-Urological Clinical Center, Key Laboratory of Urological Diseases in Gansu Province, Lanzhou University Second Hospital, Lanzhou 730030, China; 220220906021@lzu.edu.cn (G.W.); ery_mij@lzu.edu.cn (J.M.); baijt21@lzu.edu.cn (J.B.); ery_heqq@lzu.edu.cn (Q.H.)

**Keywords:** kidney stones, non-coding RNAs, kidney injury, biomarkers, therapeutic applications

## Abstract

Nephrolithiasis is a major public health concern associated with high morbidity and recurrence. Despite decades of research, the pathogenesis of nephrolithiasis remains incompletely understood, and effective prevention is lacking. An increasing body of evidence suggests that non-coding RNAs, especially microRNAs (miRNAs) and long non-coding RNAs (lncRNAs), play a role in stone formation and stone-related kidney injury. MiRNAs have been studied quite extensively in nephrolithiasis, and a plethora of specific miRNAs have been implicated in the pathogenesis of nephrolithiasis, involving remarkable changes in calcium metabolism, oxalate metabolism, oxidative stress, cell–crystal adhesion, cellular autophagy, apoptosis, and macrophage (Mp) polarization and metabolism. Emerging evidence suggests a potential for miRNAs as novel diagnostic biomarkers of nephrolithiasis. LncRNAs act as competing endogenous RNAs (ceRNAs) to bind miRNAs, thereby modulating mRNA expression to participate in the regulation of physiological mechanisms in kidney stones. Small interfering RNAs (siRNAs) may provide a novel approach to kidney stone prevention and treatment by treating related metabolic conditions that cause kidney stones. Further investigation into these non-coding RNAs will generate novel insights into the mechanisms of renal stone formation and stone-related renal injury and might lead to new strategies for diagnosing and treating this disease.

## 1. Introduction

Kidney stone disease, or nephrolithiasis, is a common condition affecting 1% to 20% of the global population [1,2]. The incidence of nephrolithiasis has been steadily increasing in recent decades, with significant implications for the well-being and financial burden of affected individuals and society as a whole [3]. Within five years of their first episode, 67% of kidney stone patients in a prospective cohort study will experience another attack [4]. Although kidney stones are common and frequently recurrent, their formation is an intricate and multifactorial process. Numerous factors, such as genetic predisposition, dietary choices, behavioral patterns, and infections, can influence the formation of kidney stones [5]. The pathophysiological mechanism of kidney stone formation is generally recognized as the precipitation, growth, and aggregation of crystal nuclei [6]. However, little is known about the cellular and molecular composition of the renal papilla, the spatial distribution of the different cell types, and their relationship to mineral deposition (such as Randall’s plaques) during stone disease, but understanding this is essential for the treatment and prevention of kidney stones [7]. Strategies to reduce the incidence of stone formation primarily include increasing fluid intake, adopting healthier lifestyles, modifying dietary habits, and administering medications that inhibit the formation of calcium oxalate (CaOx) crystals [8]. Although surgical techniques for stone removal have improved, the prevention of stone formation and recurrence remains elusive [9]. Furthermore, stone recurrence and multiple treatments raise the risk of renal damage, infection, and other problems.

The majority of the human genome is transcribed into non-coding RNAs, with less than 2% of the genome having the potential to code for proteins [10]. Non-coding RNAs encompass various subclasses, including microRNA (miRNA), long non-coding RNA (lncRNA), circular RNA (circRNA), small nuclear RNA (snoRNA), small interfering RNA (siRNA), transfer RNA (tRNA), and so on [11,12,13]. Non-coding RNAs have critical physiological regulatory roles and are linked to the development of a variety of illnesses. Numerous studies have implicated non-coding RNAs in the regulation of kidney-related diseases, such as renal tumors [11], diabetic nephropathy [14,15], renal injury and repair [13,16], and hereditary nephropathy [17]. Similarly, non-coding RNAs play an important role in the regulation of kidney stone formation and can influence the process through a variety of mechanisms, such as inflammation, oxidative stress, cellular autophagy, macrophage (Mp) recruitment, crystal adhesion, and so on. This review focuses on recent advances in the function of non-coding RNAs, specifically miRNAs and lncRNAs in kidney stones. We also discuss the potential use of non-coding RNAs as biomarkers for kidney stones and as therapeutic targets for kidney stone prevention and treatment. Furthermore, future research directions in this field are also addressed.

## 2. Differential Expression of Non-Coding RNAs in Kidney Stones

### 2.1. In Animal Models

Calcium oxalate monohydrate (COM) crystals significantly altered the overall expression profile of miRNAs in vitro, according to a study based on miRNA and mRNA microarrays, and differentially expressed miRNAs have the potential to target many genes involved in apoptosis, metabolic process control, intracellular signaling cascades, insulin signaling pathways, and type 2 diabetes [18]. Another study examined the expression profiles of miRNAs and mRNAs in the kidneys of rats with CaOx stones and discovered that 38 miRNAs and 2728 mRNAs were significantly altered [19]. According to Gene Ontology (GO) analysis, the majority of the target genes were enriched in oxidation reduction, ion transport, inflammatory response, and wound response. The Kyoto Encyclopedia of Genes and Genomes (KEGG) pathway study of these targets reveals their crucial importance in the cytokine–cytokine receptor interaction, gap junction, and chemokine signaling pathways. In a rat model of hyperoxaluria, Liu et al. found that the expression of 28 miRNAs varied in the renal tissues, and they hypothesized that insulin resistance and the phosphatidylinositol-bisphosphate 3-kinase/AKT serine-threonine kinase signaling pathway might be related to the regulation of miRNAs [20]. In addition, Lu et al. found that the miRNAs rno-miR-674-5p, rno-miR-672-5p, rno-miR-138-5p, and rno-miR-21-3p play significant roles in the miRNA-gene network of genetic hypercalciuric stone-forming rat kidneys [21].

Other non-coding RNAs, such as lncRNAs and circRNAs, have also been reported to be differently expressed in kidney stone animal models. For instance, in a study on rats with ethylene glycol (EG)-induced kidney stones, 1440 lncRNAs, 2455 mRNAs, and 145 circRNAs were found to be differentially expressed [22].

### 2.2. Patients’ Biospecimens

Distinct patterns of non-coding RNA expression have been observed in the renal tissue, urine, and blood samples of individuals diagnosed with nephrolithiasis. Liang and colleagues found that nine miRNAs, 883 mRNAs, and 1002 lncRNAs were differentially expressed in urine samples from patients with calcium oxalate kidney stones compared with healthy individuals [23]. These mRNAs were enriched in respiratory burst, mitosis regulation, and protein kinase regulator activity. The study identified five miRNAs, four mRNAs and six lncRNAs with consistent expression changes in HK-2 cells induced by NaOx. Yang et al. discovered 54 dysregulated miRNAs in urinary exosomes from CaOx stone patients and healthy controls [24]. Dysregulated miRNAs were enriched in oxidative stress, focal adhesion, and cell adhesion molecule binding. In CaOx stone patients, the expression of miR-223-3p was shown to be higher, presumably influencing stone formation [24].

Recently, Xia et al. established an immune-associated lncRNA–miRNA–mRNA competing endogenous RNA (ceRNA) network, including 10 lncRNAs, 23 miRNAs, and 20 mRNAs by analyzing differentially expressed mRNAs (DE-mRNAs) and lncRNAs in Randall’s plaques of CaOx stone patients and controls with normal renal papillary tissues [25]. The results indicated that significant expressions of ceRNAs (NEAT1, PVT1, hsa-miR23b-3p, hsa-miR-429, hsa-miR-139-5p, CCL7, and ROBO2) and infiltrating immune cells (Mps and mast cells) may be involved in the pathogenesis of kidney stones.

Although non-coding RNAs were found to be differentially expressed in both in vivo and in vitro studies of patients with CaOx stones and animal models of stones, GO and KEGG analyses alone were insufficient to determine the specific mechanism of action for these differentially expressed non-coding RNAs in kidney stones. Further research is needed to confirm the specific mechanisms of action of each differentially expressed non-coding RNA in kidney stones.

## 3. The Role and Mechanism of miRNAs in Kidney Stones

MiRNAs are short RNA molecules of 19 to 25 nucleotides in length that regulate the posttranscriptional silencing of target genes, primarily by binding to the 3′ untranslated regions of their target gene mRNAs, which either induces mRNA degradation or, more commonly, blocks mRNA translation into protein [26,27]. Emerging evidence suggests that miRNAs are critical regulators of renal development and pathophysiology [16,28,29,30,31]. We summarize the miRNAs involved in kidney stones and their regulatory mechanisms (Table 1).

### 3.1. Regulation of Calcium Metabolism

Calcium stones, which can occur as CaOx and calcium phosphate (CaP) crystals alone or in combination, are the most common type of kidney stones. In patients experiencing their first symptomatic kidney stones, approximately 93.5% of the stone composition consists of calcium stones [32]. Increased urinary excretion of calcium ions is an important factor in the formation of calcium stones [24,33]. The calcium-sensing receptor (CaSR) plays a role in inhibiting calcium reabsorption and promoting the precipitation of CaP and oxalate when stimulated by elevated serum calcium levels [34,35]. Three claudin genes, namely claudin-14, claudin-16, and claudin-19, are involved in calcium ion transport in the thick ascending limb of Henle and have been linked to renal diseases, such as hypercalciuria, kidney stones, and bone mineral loss. The regulation of the claudin-14 gene is not well understood, but recent research has shown that two miRNA molecules, miR-9 and miR-374, can directly target the 3′ untranslated region (3′UTR) of claudin-14 mRNA and induce its degradation [36]. The levels of miR-9 and miR-374 are regulated by extracellular calcium ions, and their inhibition leads to an increase in claudin-14 expression and subsequent depletion of calcium and magnesium in the kidney [37]. Activation of the CaSR decreases the expression of miR-9 and miR-374, while the inhibition of the CaSR has the opposite effect [38]. This molecular cascade of CaSR–miRNAs–claudins forms a regulatory loop that plays a crucial role in maintaining the proper balance of calcium levels in the kidney [39,40].

A 2014 epidemiological survey in China found that daily vinegar intake is strongly associated with a lower risk of nephrolithiasis among adults, with an OR of 0.36 [41]. Subsequent research found that people who regularly consume vinegar have lower calcium excretion and higher citrate levels in their urine, two critical molecules for CaOx kidney stones in humans [42]. Mechanism dissection suggested that acetate increased the acetylation of Histone H3 in renal tubular cells and stimulated the production of miRNA-130a-3p, miRNA-148b-3p, and miRNA-374b-5p by acetylating Histone H3K9 and H3K27 at their promoter regions. By suppressing the expression of sodium-dependent dicarboxylate transporter 1 (Nadc1) and claudin-14, these miRNAs can increase the excretion of citrate in the urine and decrease the excretion of calcium [37,42].

### 3.2. Metabolism of Oxalate

Changes in the concentration of urinary oxalate have been found to significantly affect the supersaturation of urinary oxalate [43,44]. Urinary oxalate is a result of both endogenous oxalate synthesis and dietary oxalate intake [45]. The liver primarily metabolizes endogenous oxalate, which is influenced by the intake of precursor substances in the diet [46]. Dysfunction in oxalate synthesis is a crucial factor in the development of CaOx stone disease and hereditary primary hyperoxaluria (PH). PH type I (PH1) is one of the most severe hyperoxaluric diseases leading to urolithiasis, nephrocalcinosis, and end-stage renal disease. Due to mutations in alanine–glyoxylate aminotransferase (AGXT) encoding alanine-glyoxylate aminotransferase (AGT), which regulates the final step of glyoxylate metabolism [47], PH1 has long been considered a monogenic disorder. However, recent reports have shown significant genotype–phenotype heterogeneity in patients with PH1, and many patients without AGXT mutations still exhibit PH1-related phenotypes [48]. Xin et al. found that miR-4660 may be a novel biomarker for mutation-negative idiopathic oxalosis by regulating the post-transcription of AGXT [49]. In their study, miR-4660 was downregulated in patients with oxalosis compared with healthy controls and epigenetically decreased the expression of AGT in human liver tissues. Moreover, the overexpression of miR-4660 led to the dysregulation of AGXT at both mRNA and protein levels by binding to the 3′UTR of AGXT [49]. A 2021 study found that miRNA-411-3p is involved in the regulation of the inhibitory effect of glycine on renal CaOx crystal depositions [50]. Glycine can reduce EG-induced CaOx crystal depositions in rat kidneys by decreasing urine oxalate and increasing urine citrate, which was achieved by downregulating Slc26a6 expression and inhibiting Nadc1 expression. Moreover, glycine decreased the protein expression of both Slc26a6 and Nadc1 by increasing the expression of miRNA-411-3p, which directly bound to the 3′ untranslated regions of Slc26a6 and Nadc1 messenger RNAs in vitro and in vivo [50].

### 3.3. Oxidative Stress and Renal Tubular Epithelial Cell (RTEC) Injury

Previous research has established a strong association between the development of kidney stones and the damage to RTECs and subsequent inflammatory responses [51,52,53,54]. It has been demonstrated that oxidative stress induced by reactive oxygen species (ROS) plays a critical role in this pathological process. Recent studies have indicated that specific miRNAs have significant implications for the oxidative stress and inflammatory responses triggered by kidney stones. For instance, Hu et al. found that patients with nephrolithiasis had considerably higher serum and urine levels of miR-155, and there was a correlation between the overexpression of miR-155 and the elevation of CRP and the reduction of eGFR. Urinary levels of miR-155 inversely correlate with urinary expression of IL-1β, IL-6, and TNF-α and positively correlate with the urinary expression of regulated upon activation, normal T-cell expressed, and secreted (RANTES, also known as C-C chemokine ligand). These findings suggest that miR-155 may play a significant role in the pathophysiology of kidney stones by modulating the expression of inflammatory cytokines [55]. Similarly, miR-155 expression was found to be upregulated in the kidneys of patients with ANCA-associated crescentic glomerulonephritis and a mouse model of crescentic glomerulonephritis, potentially contributing to the TH17 immune response and tissue damage [56]. The inhibition of miR-155 expression was shown to attenuate apoptosis and DNA damage in RTECs by increasing the expression of telomere repeat binding factor 1 and cell cycle protein-dependent kinase 12, thereby limiting telomere dysfunction and genomic DNA damage in acute kidney injury [57]. Another study revealed that miR-155-5p was significantly upregulated in HK-2 cells treated with oxalate and COM, and its inhibition resulted in a decrease in ROS production and cellular damage [58]. In a mouse model, the overexpression of miR-155-5p augmented the expression of the NOX-2 protein, indicating its potential role in enhancing oxidative stress injury caused by oxalate and COM by suppressing the expression of matrix Gla protein (MGP) [58].

Su et al. found that miR-21 promoted CaOx-induced renal tubular cell injury by targeting the peroxisome proliferator-activated receptor (PPAR)-α gene (PPARA), a key gene in fatty acid oxidation [59]. COM exposure significantly upregulated the expression of miR-21, inhibited proliferation, promoted apoptosis, and caused lipid accumulation in HK-2 cells. Furthermore, inhibition of miR-21 increased proliferation while decreasing apoptosis and lipid accumulation in HK-2 cells exposed to COM. In a glyoxylate-induced mouse model of renal calcium oxalate deposition, increased miR-21 expression, lipid accumulation, and kidney injury were observed. Further studies confirmed that PPARA is a direct target of miR-21 and that inhibition of miR-21 by miRNA antagomiR or activation of PPAR-α by fenofibrate, a selective agonist of PPAR-α, reduced renal lipid accumulation and protected the kidneys from injury [59].

Ye et al. discovered that theaflavin suppressed miR-128-3p expression and further abolished its inhibition on SIRT1 to attenuate oxidative stress in vitro [60]. In addition, miR-128-3p overexpression promoted kidney damage in nephrocalcinosis mice and partially reversed the protective effect of theaflavin treatment. Theaflavin exhibits a strong nephroprotective ability to suppress CaOx-induced kidney damage through the recovery of the antioxidant defense system regulated by the miR-128-3p/SIRT1 axis [60]. Xie et al. discovered that miR-204 blocked the activation of the extracellular signal-regulated kinase (ERK) pathway by targeting mucin 4, promoting RTEC proliferation, inhibiting ROS levels, RTEC apoptosis, and CaOx crystal formation [61]. In fact, mucin 4 activates the ERK pathway at the level of Raf-1 in contact-inhibited cells without requiring Ras activation [62]. Gan et al. found that miR-141-3p inhibited the expression of Nod-like receptor protein 3 (NLRP3) by binding to it, and the overexpression of NLRP3 reversed the protective effect of miR-141-3p on RTECs, suggesting that miR-141-3p inhibits NLRP3-mediated hemoprotein deposition and protects against CaOx crystal-induced RTEC damage [63].

Liu et al. found that the expression of miR-93-5p was significantly downregulated in urine and biopsy tissue samples from kidney stone patients, and the expression level of miR-93-5p was negatively correlated with Pknox1 [64]. When miR-93-5p was downregulated or inhibited, polyunsaturated fatty acids (PUFA) reversed the effects of decreased apoptosis and fat accumulation in a mouse kidney stone model and COM-induced HK-2 cells. The overexpression of Pknox1 counteracted the effects of the miR-93-5p upregulation on HK-2 cells induced by COM, suggesting that PUFA can prevent tubular injury caused by renal stones by modulating the miR-93-5p/Pknox1 axis [64].

### 3.4. Cell–Crystal Adhesion

Cell–crystal adhesion plays a crucial role in the formation of kidney stones. CD44, a cell surface receptor found in kidney tubular epithelial cells (HK-2), is known to promote cell-crystal adhesion [65,66]. In a study conducted by Wang et al., it was observed that exposure of HK-2 cells to COM crystals led to a decrease in the expression of miR-34a and an increase in CD44 levels [67]. Through luciferase reporter assay, it was determined that miR-34a directly targets a specific binding site in the 3′UTR of CD44. The overexpression of miR-34a inhibited CD44 expression and cell–crystal adhesion, whereas the opposite effect occurred when CD44 was overexpressed [67]. Another study found that miR-103a-3p targeted UMOD and regulated the UMOD/TRPV5 axis in oxalate-induced cells [68]. TRPV5 upregulation protected NRK-52E cells from oxalate-induced damage by increasing cell survival and decreasing CaOx adhesion. Moreover, miR-103a-3p downregulation reduced EG-induced CaOx deposition in kidney tissues in vivo by activating the UMOD/TRPV5 axis. Silencing miR-103a-3p improved CaOx deposition in rat kidneys by activating the UMOD/TRPV5 axis [68]. In a renal stone model using Sprague-Dawley rats, Fan et al. noted a decrease in the expression of miR-484 and forkhead box protein O1 (FoxO1), in addition to an increase in vitamin D receptor (VDR) expression [69]. It was supported by in vitro experiments where VDR was a target gene of miR-484 and miR-484 overexpression, or VDR inhibition reduced the cytotoxicity of RTECs and crystal attachment to RTECs, as well as reduced CaOx crystallization in vivo [69].

### 3.5. Macrophage Polarization and Metabolism

Stone formation is a complex process that involves urinary supersaturation and the immune response in the kidneys [69]. Pioglitazone, a thiazolidinedione drug, has been shown to reduce Mp infiltration and M1 Mp polarization in the kidney [70]. Similarly, rosiglitazone, another thiazolidinedione, has been found to alleviate renal tubular injury caused by oxidative stress and inflammation by inhibiting M1 Mp polarization and promoting M2 Mp polarization [71]. Chen et al. discovered that the upregulation of miR-23 in Mps is mediated by the peroxisome PPAR-γ interacting with the pre-miR-23 promoter. The overexpression of miR-23 inhibits M1 Mp polarization, reducing the formation of renal CaOx crystals and inflammatory injury [70].

Zhu et al. demonstrated that targeting the androgen receptor (AR) with the small molecule ASC-J9^®^ may function by altering Mp recruitment and M2 polarization to decrease intrarenal CaOx crystal deposition in multiple preclinical studies using multiple in vitro cell lines and in vivo mouse and rat models [72]. AR can decrease Mp colony-stimulating factor 1 (CSF-1) expression by increasing miRNA-185-5p expression to suppress M2 Mp polarization-mediated intrarenal CaOx crystal phagocytosis [72]. In a prospective study, Elshal et al. found that patients (*n* = 74) with CaOx stones had a higher expression of ARs and miRNA-185-5p compared with healthy individuals (*n* = 40), while CSF-1 expression was lower in stone formers [73]. These findings suggest that the signaling pathways of AR, miRNA, and CSF-1 are significant factors in the development of kidney stones.

Nuclear factor-E2-related factor 2 (Nrf2) plays a crucial role in inhibiting oxalate-induced oxidative damage. Dimethyl fumarate, an activator of Nrf2, has been shown to inhibit inflammation and oxidative stress induced by oxalate [74]. Total flavonoids derived from *L. christinae* have also been found to reduce oxidative stress in renal tissue by activating the Nrf2/antioxidant response element (ARE) pathway [75]. The overexpression of the circadian gene BMAL1 activates the Nrf2/HO-1 pathway, mitigating the oxidative damage caused by oxalate [76]. According to one study, sulforaphane can reduce kidney damage and CaOx crystal deposition in the kidneys induced by CaOx nephrocalcinosis. In vitro and in vivo, sulforaphane may increase M2 Mp polarization and prevent CaOx nephrocalcinosis-induced inflammatory damage to RTECs via the Nrf2-miR-93-TLR4/IRF1 pathway [77]. Nrf2 exhibited a positive transcriptional activation of miR-93-5p, which targets toll-like receptor 4 (TLR4) and interferon regulatory factor 1 (IRF1) mRNA. Furthermore, suppressed miR-93-5p expression partially reversed Nrf2-dependent TLR4/IRF1 downregulation [77]. Moreover, the elevated expression of ceRNAs such as NEAT1, PVT1, hsa-miR-23b-3p, hsa-miR-429, hsa-miR-139-5p, CCL7, and ROBO2, along with the presence of infiltrating immune cells like Mps and mast cells, may be associated with the development of kidney stones [25].

### 3.6. Cellular Autophagy

Shi et al. conducted a study in which they investigated the protective effects of exosomes enriched with miR-20b-3p, derived from adipose-derived stromal cells (ADSCs), on rats with EG-induced hyperoxaluria [78]. The researchers found that co-culturing RTECs with exosomes enriched with miR-20b-3p effectively reduced oxalate-induced cellular autophagy and inflammatory responses, as demonstrated in cellular experiments. This reduction was achieved by inhibiting the expression of autophagy-related gene 7 (ATG7) and TLR4. These findings suggest that miR-20b-3p-enriched exosomes derived from ADSCs have the potential to protect individuals with kidney stones by suppressing autophagy and inflammatory responses [78].

### 3.7. Apoptosis

One study conducted by Wang et al. found that the presence of oxalate in RTECs led to apoptosis and injury [53]. Previous research has shown that miR-30c is involved in regulating apoptosis and proliferation in these cells [79]. Wang et al. also discovered that increasing the expression of miR-30c-5p in HK-2 cells resulted in a reversal of apoptosis and a decrease in crystal formation. This increase in miR-30c-5p also led to a reduction in LDH, MDA, and ROS levels. Furthermore, the overexpression of miR-30c-5p resulted in an upregulation of superoxide dismutase (SOD), catalase, and matrix metalloproteinase (MMP) levels induced by sodium oxalate. Further investigations revealed that miR-30c-5p directly regulates autophagy-related 5 (ATG5), and the suppressive effects of miR-30c-5p mimics on cytotoxicity and crystal cell adhesion were counteracted by ATG5 [80].

**Table 1:** Summary of miRNAs involved in kidney stones.

MiRNA	Expression in Patients	Function	Upstream Effector	Target	Model/Sample	Manipulation	Refs
miR-9, miR-374	NA	Protective	CaSR	claudin-14	Mouse MKTAL cells, mouse TAL cells, human HEK293 cells, mice	In vitro: mimic In vivo: anti-miR	[36,37,38,39,40]
miRNA-130a-3p, miRNA-148b-3p, miRNA-374b-5p	NA	Protective	Histone H3K9 and H3K27	Nadc1 and claudin-14	HK-2 cells, rats	In vitro: mimic and anti-miR In vivo: anti-miR	[37,42]
miR-4660	Down	Pathogenic	NA	AGXT	Human serum, human liver tissues, HepG2 and L02 cell lines	In vitro: mimic and anti-miR	[49]
miRNA-411-3p	NA	Protective	glycine	Slc26a6 and Nadc1	HK-2 and NRK-52E cells, rats	In vitro: mimic and anti-miR In vivo: mimic and anti-miR	[50]
miR-155-5p	Up	Pathogenic	NA	MGP	Human serum and urinary, HK-2 cells, mouse	In vitro: anti-miR In vivo: mimic and anti-miR	[55,58]
miR-21	Up	Pathogenic	NA	PPARA	Human urine, HK-2 cells, mouse	In vitro: anti-miR In vivo: anti-miR	[59]
miR-128-3p	NA	Pathogenic	Theaflavin	SIRT1	HK-2 cells, mouse	In vitro: mimic and anti-miR In vivo: mimic and anti-miR	[60]
miR-204	NA	Protective	NA	mucin 4	HK-2 cells	In vitro: mimic and anti-miR	[61]
miR-141-3p	NA	Protective	NA	NLRP3	HK-2 cells	In vitro: mimic	[63]
miR-93-5p	Down	Protective	PUFA	Pknox1	Urine samples of patients, biopsy tissue samples from patients, mice, HK-2 cells	In vitro: mimic and anti-miR In vivo: anti-miR	[64]
miR-93-5p	NA	Protective	Nrf2	TLR4 and IRF1	BMDMs, TECs, mouse,	In vitro: mimic and anti-miR In vivo: anti-miR	[77]
miR-34a	NA	Protective	NA	CD44	HK-2 cells, mice	In vitro: mimic and anti-miR In vivo: anti-miR	[67]
miR-103a-3p	NA	Pathogenic	NA	UMOD	NRK-52E cells, rat	In vitro: mimic and anti-miR In vivo: anti-miR	[68]
miR-484	NA	Protective	NA	VDR	RTECs, rat	In vitro: mimic and anti-miR In vivo: anti-miR	[69]
miR-23	NA	Protective	PPAR-γ	IRF1/Pknox1	BMDMs, mice	In vitro: mimic and anti-miR In vivo: mimic	[70]
miRNA-185-5p	Up	Pathogenic	AR	CSF-1	human plasma samples; HK-2 and HKC-8 cells, mice	In vivo: mimic	[72,73]
miR-20b-3p	Down	Protective	NA	ATG7, TLR4	Urine of patients, NRK-52E cells, rat	In vitro: mimic In vivo: mimic	[78]
miR-30c-5p	NA	Protective	NA	ATG5	HK-2 cells	In vitro: mimic and anti-miR	[80]

CaSR, calcium-sensing receptor; Nadc1, sodium-dependent dicarboxylate transporter 1; TAL, thick ascending limb; AGXT, alanine–glyoxylate aminotransferase; HepG2, human liver tumor cell line; L02, normal cell line; MGP, matrix Gla protein; PPARA, peroxisome proliferator-activated receptor (PPAR)-α gene; NLRP3, Nod-like receptor protein 3; PUFA, Polyunsaturated fatty acid; Nrf2, nuclear factor erythroid 2-related factor 2; BMDMs, bone marrow-derived macrophages; TECs, renal tubular epithelial cells; UMOD, uromodulin; VDR, vitamin D receptor; RTECs, renal tubular epithelial cells; IRF1, interferon regulatory factor 1; AR, androgen receptor; CSF-1, colony-stimulating factor 1; ATG7, autophagy-related gene 7; TLR4, toll-like receptor 4; ATG5, autophagy-related 5.

## 4. The Role and Mechanism of lncRNAs in Kidney Stones

LncRNAs are longer than 200 nucleotides in length [81]. They are present in both the cell nucleus and cytoplasm but are not translated into functional proteins. Human GENCODE statistics indicate that the human genome has more than 16,000 lncRNA genes, whereas other estimates approach 100,000 human lncRNAs [82,83]. Unlike miRNAs, which only act post-transcriptionally, lncRNAs can act both transcriptionally and post-transcriptionally by interacting with DNA, RNA, and proteins [84]. We summarize the possible roles of lncRNAs in kidney stones (Table 2). However, studies on the regulatory roles of lncRNAs in the pathogenesis of kidney stones are limited and warrant further investigation.

### 4.1. Function as miRNA Sponges

**Figure 1:**
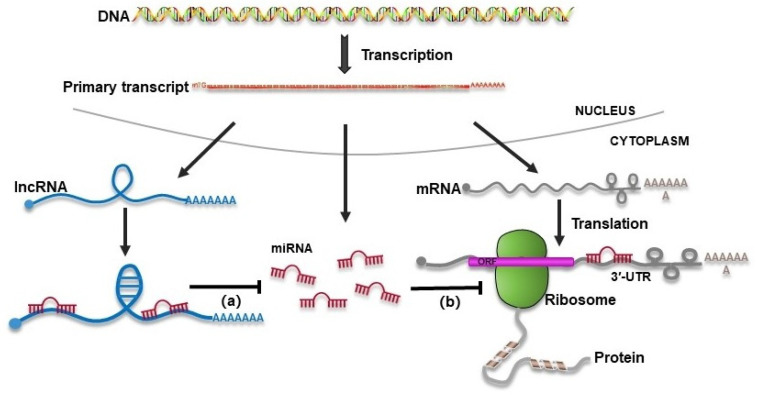
Common mechanisms of lncRNA as ceRNA bind miRNAs to regulate the expression of protein-coding mRNAs. (**a**) High expression of lncRNAs increases intracellular lncRNA transcripts, which compete to bind more miRNA molecules, leading to a decrease in the number of miRNA molecules. (**b**) The decreased number of miRNAs weakens the inhibition of protein-coding mRNA expression by miRNAs, resulting in the corresponding proteins increasing with the increased expression level of lncRNAs.

Some lncRNAs reduce the inhibitory effect of miRNAs on target mRNAs by acting as molecular sponges to competitively bind miRNAs (Figure 1). Song et al. conducted a study that investigated the regulatory role of lncRNA LINC00339 in the expression of NLRP3 and cellular autophagy in HK-2 cells treated with COM [85]. The study found that LINC00339 upregulated NLRP3 expression by acting as a sponge for miR-22-3p, thereby promoting cellular autophagy. In COM-treated HK-2 cells, the increased expression of LINC00339 and the decreased expression of miR-22-3p led to the activation of NLRP3 inflammasome and enhanced cellular thermophilia. Conversely, the overexpression of miR-22-3p and knockdown of NLRP3 had the opposite effect, inhibiting NLRP3 inflammasome activation and cellular pyrolysis. Another study by Liu et al. demonstrated that the upregulation of lncRNA H19 was positively correlated with the expression levels of High Mobility Group Protein 1 (HMGB1), TLR4, and NF-κB in mouse models of CaOx renal calcification [86]. The downregulation of H19 suppressed the expression of HMGB1, TLR4, and NF-κB and inhibited CaOx renal stone-induced RTEC injury, NADPH oxidase, and oxidative stress. The study suggested that H19 may facilitate CaOx renal calcification through oxidative stress-induced RTEC injury and that miR-216b may mediate the effect of H19 on HMGB1. Additionally, Li et al. identified a novel lncRNA, HOXA11-AS, which was upregulated in CaOx kidney stones [87]. HOXA11-AS inhibited proliferation, promoted apoptosis, and exacerbated cellular damage in HK-2 cells exposed to COM. The study revealed that HOXA11-AS modulated the role of monocyte chemotactic protein 1 (MCP-1) in the HK-2 cell CaOx nephritis model through its interaction with miR-124-3p. Furthermore, Lv et al. demonstrated the involvement of lncRNA XIST in kidney stone pathogenesis. XIST was found to interact with miR-223-3p and the NLRP3/Caspase-1/IL-1β pathway, thereby modulating inflammatory responses, ROS production, and kidney stone development [88]. Another study by Li et al. investigated the role of lncRNA-ATB in COM-induced cell injury, apoptosis, proliferation inhibition, and epithelial–mesenchymal transition (EMT) in HK-2 cells [89]. The study suggested that lncRNA-ATB played a role in CaOx crystal-induced kidney injury through its interaction with miR-200s. Additionally, the expression of LINC01197 and Sirtuin 3 (SIRT3) was found to be dysregulated in patients with kidney stones [90]. The knockdown of LINC01197 enhanced cell adhesion and apoptosis induced by CaOx, while overexpression of SIRT3/FOXO1 enhanced the expression of LINC01197. The study concluded that LINC01197 inhibited the formation of kidney stones induced by calcium oxidation through the regulation of the miR-516b-5p/SIRT3/FOXO1 signaling pathway [90].

A key mechanism of action for many miRNAs/lncRNAs is that lncRNAs act as molecular sponges, competing to bind miRNAs and, thereby, upregulate miRNA target gene expression. However, this hypothesis is controversial and only applicable under very particular circumstances. Based on recent research modeling transcriptome-wide binding-site abundance, most individual transcripts’ physiological variations in expression do not appear to affect miRNA activity [91].

**Table 2:** Summary of lncRNAs involved in kidney stones.

LncRNAs	Expression in Patients	Function	Target Pathway	Model	Refs
LINC00339	NA	Pathogenic	miR-22-3p/NLRP3	HK-2 cells	[85]
H19	Up	Pathogenic	miR-216b/HMGB1/TLR4/NF-kB	Randall’s plaques, HK-2 cells, mouse	[86]
HOXA11-AS	NA	Pathogenic	miR-124-3p/MCP-1	HK-2 cell, mouse	[87]
XIST	NA	Pathogenic	miR223/NLRP3/Caspase-1/IL-1β	HK-2 cell, mouse	[88]
lncRNA-ATB	NA	Pathogenic	miR-200 family	HK-2 cell	[89]
LINC01197	Down	Protective	miR-516b-5p/SIRT3/FOXO1	Renal tissues of patients, HK-2 cell	[90]
CHCHD4P4	NA	Pathogenic	NA	HK-2 cells, mouse	[92]
OLMALINC	NA	Pathogenic	OCT4/BMP2	hRIFs	[93]

NLRP3, Nod-like receptor protein 3; HMGB1, High Mobility Group Protein 1; SIRT3, Sirtuin 3; OCT4, octamer binding transcription factor 4; BMP2, bone morphogenetic protein type 2; hRIFs, human renal interstitial fibroblasts.

### 4.2. Facilitating Fibrosis

Zhang et al. discovered that a total of 376 mouse lncRNAs were differentially expressed between glyoxylate-exposed and healthy mouse kidneys using microarray technology and bioinformatics analysis [92]. Using the Basic Local Alignment Search Tool (BLAST), they identified 15 mouse and human lncRNA homologs, including AU015836 and CHCHD4P4. Subsequent investigations revealed that CHCHD4P4 plays a role in inhibiting cell proliferation and promoting epithelial–mesenchymal transition in the context of renal injury and fibrosis induced by CaOx crystallization and deposition. Furthermore, the researchers found that silencing CHCHD4P4 led to a reduction in renal injury and fibrosis [92]. The formation of renal CaOx stones at Randall’s plaque has been recognized as a stationary occurrence, although the underlying mechanism remains unclear [93]. Osteoblast-like cells have been shown to significantly influence the formation of regenerative periosteum (Randall’s plaque), and the transcription factor OCT4 has been found to facilitate the conversion of differentiated cells into osteoblasts. RNA immunoprecipitation and RNA analyses demonstrated that the lncRNA OLMALINC directly interacts with OCT4 and enhances its stability by disrupting the ubiquitination process. Furthermore, OLMALINC was upregulated in Randall’s plaque fibroblasts visualized by fluorescence in situ hybridization (FISH), and a positive correlation between OLMALINC and OCT4 in Randall’s plaque was observed. It has been proposed that OLMALINC contributes to the development of an osteoblast-like phenotype in fibroblasts, thereby contributing to the formation of Randall’s plaques [93]. Xia et al. conducted a study that demonstrated a notable enrichment of differentially expressed mRNAs (DE-mRNAs) associated with lncRNAs in extracellular matrix tissues and extracellular matrices containing collagen, which are closely associated with renal interstitial fibrosis [25].

In conclusion, emerging studies have shown that miRNAs and lncRNAs play different roles in renal stone formation and stone-associated renal injury. The HK-2 cells used in most of the cited studies were derived from proximal tubular cells, but stone formation occurs mostly in the medullary region of the loop of Henle and in the collecting duct and renal papilla. According to the free-particle mechanism, crystals of stones form in the renal tubules, move with the urine, aggregate, and plug the loop of Henle and terminal collecting ducts. In addition, it has been suggested that crystal–cell interactions are likely to be an important factor in stone formation and that oxidative stress in the proximal tubules might induce lipid peroxidation and damage to the brush border membrane at this level of the nephron. Released membrane fragments and vesicles containing phospholipids are considered important as promoters of both calcium phosphate (CaP) and calcium oxalate (CaOx) precipitation at supersaturation levels lower than those otherwise required for crystallization [5]. However, it must be recognized that the interaction of crystals with renal tubular epithelial cells is not sufficient to explain the complex mechanisms of stone formation and that more factors and physiological mechanisms are involved in the formation of renal stones.

## 5. Non-Coding RNAs as Biomarkers for Kidney Stone

Some non-coding RNAs have altered expression profiles in kidney stones and may have the potential as biomarkers to aid in the diagnosis of kidney stones and to assess the extent of kidney damage and recovery after treatment [23,24,73,94]. However, none of the non-coding RNAs are currently used as routine tests for kidney stone patients. Although many of the above miRNAs and lncRNAs have been investigated in the mechanism of action associated with kidney stones, most of them have only been reported in isolated literature reports and lack expression data in human tissues or biological samples. Therefore, further validation is needed to determine whether they have the same expression trends in tissues or biological samples from kidney stone patients as in animal or cellular experiments. Six miRNAs (miR-4660, miR-155-5p, miR-21, miR-93-5p, miRNA-185-5p, and miR-20b-3p) and two lncRNAs (H19 and LINC01197) were significantly differentially expressed in kidney stone patients, and their mechanisms of action were elucidated in cell and animal experiments and may be the most promising biomarkers.

MiRNAs are exceedingly stable and may be detected and analyzed in a range of physiological fluids, including plasma and urine [95]. For example, miR-223-3p was more highly expressed in urinary small Extracellular Vesicles (sEVs) from CaOx stone patients [24]. In a prospective investigation using plasma samples, miRNA-185-5p expression was significantly higher in patients with CaOx stones (*n* = 74) than in healthy subjects (*n* = 40) [73]. Five miRNAs (miR-6796-3p, miR-30d-5p, miR-3192-3p, miR-518b, and miR-6776-3p) and six lncRNAs (lnc-TIGD1L2-3, lnc-KIN-1, lnc-FAM72B-4, lnc-EVI5L-1, lnc-SERPINI1-2, and lnc-MB-6) were differentially expressed in both CaOx patients’ urine and NaOx-induced HK-2 cells [23]. Some lncRNAs can be used as tumor biomarkers [96,97,98,99,100,101]. Similarly, lncRNAs may also have the potential to be kidney stone biomarkers. For instance, LncRNA SBF2-AS1 and FENDRR-19 levels were higher in renal stone patients (*n* = 60) compared with healthy volunteers (*n* = 30) and were significantly upregulated after extracorporeal shock wave lithotripsy (ESWL) treatment in urine [94]. However, whether these miRNAs and lncRNAs actually play relevant roles in the mechanism of action of kidney stones needs further validation and elucidation.

Although the preceding studies showed that non-coding RNAs have the potential to be used as biomarkers for kidney stones, their specificity for kidney stones needs to be considered. Some non-coding RNAs have been linked to not only kidney stones but also other disorders. For example, serum miR-223-3p level is also significantly increased in patients with myotonic dystrophy type 1 [102] or unstable coronary artery disease [103]. Moreover, non-coding RNAs may have different biological significance in different body fluids and blood fractions, and there may be differences in the non-coding RNA changes caused by different types of stones.

## 6. Potential Therapeutic Applications

Interfering with the expression of critical pathways using non-coding RNA mimics or inhibitors has the potential to reduce stone formation or inhibit stone development and associated kidney damage. A few miRNAs are already in clinical trials for other diseases [104]. However, there are currently no miRNAs in clinical trials for kidney stones. SiRNAs have shown promise in the therapy of multiple diseases, such as cardiovascular disease [105], hereditary transthyretin amyloidosis [106], and heterozygous familial hypercholesterolemia [107]. 

For example, the inhibition of liver lactate dehydrogenase activity by RNA interference (RNAi) reduces urinary oxalate excretion in mouse models of PH1 [108]. PH is a rare genetic disorder characterized by excessive production of oxalate, leading to the formation of kidney stones, renal calcification, renal failure, and systemic oxalosis [109]. In November 2020, lumasiran (Oxlumo™), a subcutaneous siRNA, was approved in the USA for the treatment of adult and pediatric patients with PH1 [110]. Lumasiran targets mRNA for hydroxyacid oxidase 1 (HAO1) in hepatocytes via RNAi, thereby reducing the levels of the glycolic acid oxidase (GO) enzyme, which reduces the availability of glyoxylate, a substrate for oxalic acid production [111,112]. A double-blind phase 3 clinical trial in 39 patients (lumasiran group = 26, placebo group = 13) with PH1 showed that lumasiran reduced urinary oxalate excretion and the majority of patients receiving lumasiran had normal or near-normal levels after 6 months of treatment [113]. Since many metabolic diseases are associated with the development of kidney stones, siRNA therapy targeting these diseases may be able to reduce stone formation while treating the underlying disease; however, there is a lack of research to support this. 

Notably, developing RNA-based therapies to prevent kidney stone formation and treat stone-related kidney injury is extremely challenging. First, stimulation-induced phenotypic cells and animal models in artificial environments do not fully mimic the complicated pathways underlying human kidney stone development. Second, RNA-based drugs represented by siRNAs, which are mainly used to regulate gene expression in immune cells and hepatocytes to immunize against infections and to treat metabolic diseases, are non-specific and cannot selectively target specific tissues [114]. Moreover, naked RNA does not easily cross negatively charged hydrophobic cell membranes and is degraded by nuclease and cleared by the kidney and reticuloendothelial system in the absence of modifications [115,116]. Therefore, future studies could focus on modifying or packaging potential therapeutic RNA molecules into selective delivery vehicles for preferential delivery to kidney cells.

## 7. Summary and Outlook

The high prevalence and frequent recurrence of renal calculi have significant financial implications for patient healthcare and increase the risk of kidney injury. Consequently, there has been a concerted effort within the field of urology to identify effective methods for preventing stone formation and recurrence. A promising avenue of research involves investigating the role of non-coding RNAs, particularly miRNAs and lncRNAs, in kidney stones. By studying this relationship, we can gain a deeper understanding of the pathophysiological mechanisms underlying stone formation and the associated renal injury. This knowledge may ultimately lead to new strategies for preventing stones and mitigating the renal damage they cause. The study of epigenetic mechanisms in organisms has been expanding in both scope and depth, revealing the significance of epigenetics beyond our previous understanding. Over the past decade, researchers have increasingly focused on the role of non-coding RNAs in kidney stones, identifying potential targets for intervention in stone treatment. While animal studies have shown promising results in reducing stone formation and stone-induced kidney injury through intervention with these miRNAs and lncRNA targets, there is a lack of safety and efficacy evaluations in clinical patients.

## Data Availability

Not applicable.
